# Pangenome analysis and virulence profiling of *Streptococcus intermedius*

**DOI:** 10.1186/s12864-021-07829-2

**Published:** 2021-07-09

**Authors:** Dhiraj Sinha, Xifeng Sun, Mudra Khare, Michel Drancourt, Didier Raoult, Pierre-Edouard Fournier

**Affiliations:** 1 Aix-Marseille University, IRD, AP-HM, SSA, VITROME, IHU Méditerranée Infection, 19-21 Bd Jean Moulin, 13005 Marseille, France; 2 IHU Méditerranée Infection, Marseille, France; 3 Aix-Marseille University, IRD, AP-HM, MEPHI, IHU Méditerranée Infection, Marseille, France

**Keywords:** *Streptococcus intermedius*, *Streptococcus anginosus* group, Infection, Virulence, Comparative genomics, Whole genome sequencing, Pangenome analysis

## Abstract

**Background:**

*Streptococcus intermedius*, a member of the *S. anginosus* group, is a commensal bacterium present in the normal microbiota of human mucosal surfaces of the oral, gastrointestinal, and urogenital tracts. However, it has been associated with various infections such as liver and brain abscesses, bacteremia, osteo-articular infections, and endocarditis. Since 2005, high throughput genome sequencing methods enabled understanding the genetic landscape and diversity of bacteria as well as their pathogenic role. Here, in order to determine whether specific virulence genes could be related to specific clinical manifestations, we compared the genomes from 27 *S. intermedius* strains isolated from patients with various types of infections, including 13 that were sequenced in our institute and 14 available in GenBank.

**Results:**

We estimated the theoretical pangenome size to be of 4,020 genes, including 1,355 core genes, 1,054 strain-specific genes and 1,611 accessory genes shared by 2 or more strains. The pangenome analysis demonstrated that the genomic diversity of *S. intermedius* represents an “open” pangenome model. We identified a core virulome of 70 genes and 78 unique virulence markers. The phylogenetic clusters based upon core-genome sequences and SNPs were independent from disease types and sample sources. However, using Principal Component analysis based on presence/ absence of virulence genes, we identified the *sda* histidine kinase, adhesion protein *LAP* and capsular polysaccharide biosynthesis protein *cps4E* as being associated to brain abscess or broncho-pulmonary infection. In contrast, liver and abdominal abscess were associated to presence of the fibronectin binding protein *fbp54* and capsular polysaccharide biosynthesis protein *cap8D* and *cpsB*.

**Conclusions:**

Based on the virulence gene content of 27 *S. intermedius* strains causing various diseases, we identified putative disease-specific genetic profiles discriminating those causing brain abscess or broncho-pulmonary infection from those causing liver and abdominal abscess. These results provide an insight into *S. intermedius* pathogenesis and highlights putative targets in a diagnostic perspective.

## Introduction

*Streptococcus intermedius* belongs to the *S. anginosus* group (SAG) that also includes *S. constellatus* and *S. anginosus* [1]. It is part of the normal oral cavity and upper respiratory tract floras, as well as those of the gastrointestinal and female urogenital tracts [2–5]. This bacterium was first described by Guthof in 1956 after being isolated from dental abscesses [6]. *S. intermedius* may also cause human infections, usually monomicrobial, including purulent abscesses of the liver, lungs, psoas, spine and/or central nervous system, and infective endocarditis [7]. Over the years, the role of *S. intermedius* in human infections has increasingly been reported. Patients with invasive *S. intermedius* infections were described to cause significantly longer hospital stays and higher mortality than patients with other *S. anginosus* group infections, suggesting that identifying this species might be important for the management of patients [8].

Various putative virulence factors have been described for *Streptococcus intermedius*, among which the ability to form biofilms to protect itself from antibiotics and the host immune system [9], the production of hydrolytic enzymes, including both glycosaminoglycan-degrading enzymes, such as hyaluronidase and chrondroitin sulphate depolymerase, and glycosidases, such as α- N- acetylneuramidase (sialidase), β-D-galactosidase, N-acetyl-β-D-glucosaminidase and N-acetyl-β-D-galactosaminidase, which allow *S. intermedius* to grow on macromolecules found in host tissue [10]; a cytotoxin, intermedilysin *(ILY)*, that can directly damage host tissues and immune defense cells and participate in bacterial pathogenicity; and the surface protein antigens I/II that are involved in adhesion to fibronectin and laminin, which is an important step in the pathogenesis of endocarditis and abscess formation [11].

The development of high throughput nucleic acid sequencing technologies has enabled observing variations of the genetic repertoire among strains of a given bacterial species. Our present study analysis aimed at describing the genetic diversity and pathogenesis substratum of *S. intermedius*. Twenty-seven genomic sequences from *S. intermedius* strains, including 13 newly sequenced from our laboratory and 14 from public databases, were used for pan-genomic analysis. Predicted genes were compared among strains to determine the size of the core and dispensable gene pools, the pangenome, the gain/loss of putative virulence determinants, and to identify genomic islands.

### Accession numbers

The 13 genome sequences determined in this study were deposited in GenBank and their accession numbers are listed in Table 1.

**Table 1:** Genomic characteristics of the 27 studied *S. intermedius* strains

	Isolation sources	Size (bp)	CDS	tmRNA	tRNAs	rRNA operons	GC%	Avg length CDS (bp)	Coding %	Plasmid	Prophages	Accession number	Country
G1562	Brain abscess	2,052,377	1954	1	50	4	37.3	926	88.173	no	2	UENI00000000.1	France
G1563	Brain abscess	1,890,941	1814	1	50	4	37.7	911	80.54	no	1	UEND00000000.1	France
G1564	Brain abscess	1,887,926	1857	1	50	3	37.7	886	87.192	no	2	UENF00000000.1	France
G1565	Brain abscess	1,972,424	1905	1	50	3	37.6	905	87.4	no	1	UENG00000000.1	France
G1566	Brain abscess	1,897,154	1848	1	56	4	37.6	904	88.077	no	1	UICY00000000.1	France
G1567	Brain abscess	1,897,330	1849	1	50	4	37.6	903	88.033	no	1	UENA00000000.1	France
G1568	Brain abscess	1,898,090	1850	1	51	3	37.6	903	88.051	no	1	UENB00000000.1	France
TYG1620	Brain abscess	2,006,877	1957	1	61	4	37.6	893	87.107	no	3	AP014880.1	Japan
BA1	Brain abscess	1,965,880	1949	1	64	5	37.7	876	86.87	no	4	ANFT00000000.1	US
FDAARGOS_233	Abdominal abscess	1,914,006	1832	1	61	12	37.7	908	86.927	no	1	CP020433.2	US
G1552	Abdominal abscess	1,942,413	1883	1	51	3	37.6	899	87.182	no	1	UENJ00000000.1	France
G1553	Abdominal abscess	1,974,268	1907	1	59	4	37.6	909	87.782	no	2	NZ_UZBH00000000.1	France
G1556	Abdominal abscess	1,850,828	1805	1	56	6	37.8	901	87.87	no	1	UENK00000000.1	France
G1557	Abdominal abscess	1,898,297	1834	1	47	4	37.7	905	87.463	no	2	UENH00000000.1	France
B196	Broncho-pulmonary abscess, septic arthritis, osteomyelitis, pyomyositis	1,996,214	1884	1	61	4	37.6	924	87.175	no	1	CP003857.1	Canada
C270	Broncho-pulmonary abscess	1,960,728	1842	1	61	4	37.6	925	86.876	no	1	CP003858.1	Canada
G1554	Broncho-pulmonary abscess	1,886,668	1782	1	54	4	37.8	924	87.317	no	1	UENC00000000.1	France
631_SCON	Broncho-pulmonary abscess	1,968,891	1856	1	55	3	37.8	930	87.666	no	1	JUZI00000000.1	US
KCOM 1545	Endodontic infection	1,908,201	1835	1	32	6	37.6	910	87.514	no	1	CP012718.1	South Korea
F0413	Endodontic infection	1,921,347	1913	1	62	5	37.6	883	87.891	no	2	AFXO00000000.1	US
G1555	Bone abscess	2,003,390	1971	1	55	4	37.5	888	87.382	no	2	UENE00000000.1	France
LC4	Liver abscess	1,914,382	1832	1	41	3	37.8	918	87.889	no	1	PNRP00000000.1	China
30,309	Liver abscess	1,956,646	1864	1	47	3	37.5	920	87.648	no	1	PNRI00000000.1	China
32,811	Liver abscess	1,971,034	1930	1	47	3	37.7	896	87.751	no	1	PNRH00000000.1	China
ATCC 27,335	NA	1,951,449	1871	1	62	4	37.7	908	87.091	no	1	ATFK00000000.1	US
JTH08	NA	1,933,610	1840	1	68	4	37.7	915	87.108	no	1	AP010969.1	Japan
SK54AJKN0	NA	1,910,718	1832	1	29	3	37.6	917	87.941	no	1	AJKN00000000.1	US

*NA* not available, *bp* base pairs, *CDS* coding sequences, *prophages* Number of prophages

## Materials and methods

### Extraction and genome sequencing

The genomic DNA (gDNA) of each studied *S. intermedius* strain was extracted in two steps: a mechanical treatment was first performed using acid-washed glass beads (G4649-500 g Sigma) and a FastPrep BIO 101 instrument (Qbiogene, Strasbourg, France) at maximum speed (6.5) for 90 s. following a 2-hour lysozyme incubation at 37 °C, DNA was extracted using an EZ1 biorobot and the EZ1 DNA Tissue kit (Qiagen, Hilden, Germany). The elution volume was 50µL. Genomic DNA was quantified using the Qubit assay (Life technologies, Carlsbad, CA, USA).

The gDNAs were sequenced using a MiSeq sequencer with the Paired-End strategy and the Nextera XT library kit (Illumina, Inc, San Diego, CA, USA). The Paired-End library was prepared using input solutions of 1 ng gDNAs. The gDNAs were fragmented at the tagmentation step. Then, limited cycle PCR amplification (12 cycles) completed the tag adapters and introduced dual-index barcodes. After purification on AMPure beads (Life technologies, Carlsbad, CA, USA), the libraries were normalized according to the Nextera XT protocol (Illumina). Normalized libraries were pooled for sequencing on a MiSeq sequencer (Illumina). Automated cluster generation and paired-end sequencing with dual index reads was performed in a single 39-hour run in a 2 × 250 bp format. The numbers of paired-end reads were summarized in Table 2. The paired-end reads were trimmed and filtered according to the read qualities.

**Table 2:** Genome sequencing details of the 13 *S. intermedius* strains from our study

Strain	Extraction	Sequencing data	
	**DNA concentration ng/µL**	**Index**	**Paired end reads**
G1552	8.83	4.1	218,834
G1553	5.06	7.45	397,506
G1554	4.66	2.44	130,063
G1555	12.2	5.74	306,282
G1556	11.67	6.83	364,005
G1557	13.37	7.7	410,636
G1562	2.63	4.62	943,724
G1563	1.03	7.73	1,579,852
G1564	0.31	7.51	1,536,166
G1565	0.7	3.91	798,461
G1566	0.65	3.48	712,214
G1567	1.05	5.42	1,107,754
G1568	0.77	6.19	1,265,488

### Genome assembly, annotation and comparison

After sequencing, the obtained reads were assembled using the A5 software [12] with default parameters and then contigs were compared to NCBI using BLASTn to remove contaminations. Then, the online tool Fasta dataset joiner (http://users-birc.au.dk/biopv/php/fabox/fasta_joiner.php) was used to merge sequences into a single molecule. The Mauve software was used for multiple genomic sequence alignment [13]. Genes were annotated using the Prokka software with default parameters [14] in which the similarity e-value cut-off is 0.000001 and the minimum contig size is 200 bp. This pipeline also includes several other tools like Aragorn for tmRNA detection, Barnap to count rRNAs and Prodigal to identify coding sequences. To estimate the mean level of sequence similarity at the genome level among studied strains, we used the OrthoANI [15] and Genome-to-Genome Distance Calculator (GGDC) [16] softwares, with the following respective threshold values of 95–96 and 70 %.

### Phylogenetic analysis

A 16 S rRNA-based phylogenetic analysis of the 27 studied *S. intermedius* strains was performed using the MEGA 7 software [17]. For constructing the phylogenetic tree, the following options were used: Maximum Likelihood method; Kimura 2-parameter model for substitution model; uniform rates among sites; partial deletion option for gaps/missing data; 1000 bootstrap replicates.

Using genomic sequences and the Roary program [18], a clustered heatmap of core genes was drawn on the basis of the presence/absence approach [18]. We also detected SNPs with the snp-sites program [19] from the core genome alignment and drew a phylogenetic tree with CGEwebface [20].

### Virulence factor analysis

Virulence-associated genes were detected by comparing studied genomic sequences with the virulence factor database (VFDB) [21] and sequences described in recent publications [22]. The BLASTp search was performed using the threshold scores reported by Olson et al.: 35 % identity and highest scoring pair length of 50 % [22]. Additionally, we reviewed the literature to identify the proteins involved in interactions with the host [10, 23, 24]. A principal component analysis was performed using the XLSTAT program (Data Analysis and Statistical Solution for Microsoft Excel, Addinsoft, Paris, France 2017) in which the Fisher’s least significant difference (LSD, α = 0.005) and Pearson’s correlation coefficients were used, to detect any association of virulence-associated genes with specific clinical conditions.

### Core and pan-genome analysis

Get_homologue [25] was used to reveal orthologous genes among *S. intermedius* strains, using the following parameters: minimal coverage (-C) 40 %, minimum identity (-S) 50 %, minimum e-value (-E) 1e-05. Sequence similarity searches and clustering of coding sequence (CDS) from the 27 genomes were performed using pairwise BLASTp and OrthoMCL algorithms [26]. Sequential inclusion of all possible combinations of up to 27 strains were simulated and fitted by regression analysis [27] of the amount of conserved genes and of strain-specific genes. This allowed to estimate and extrapolate the sizes of core- and pan-genomes. Roary [18] was also used, with default parameters, to confirm the reliability of the obtained pan-genome analysis results (identity percent ≥ 70 %, coverage ≥ 70 %) and to generate the core genome alignment.

### Functional classification of orthologous cluster analysis

The Clusters of Orthologous Groups (COGs) database was used to identify gene functions [28] using BLASTP (E-value 1e^− 03^, coverage 0.7 and identity percent 30 %).

A circular comparison of genomes was obtained using the online GView Server (https://server.gview.ca/) with *S. intermedius* strain ATCC 27,335 as reference genome [29]. ResFinder and the ARG-ANNOT database were used to search antibiotic resistance-related markers [30, 31]. The presence of CRISPR repeats and prophages was predicted using the CRISPRFinder [32] and PHASTER softwares, respectively [33].

## Results and discussion

### Strain characterization

The 27 studied *S. intermedius* strains originated from China, Canada, South Korea, US, Japan and France. The patients’ data was not available for some strains. The 13 French strains (G1552-G1557 and G1562-G1568, Tables 1 and 2) were isolated in our laboratory from patients with various infections (Table 2), from August 2014 to November 2016, on 5 % sheep blood-enriched Columbia agar (BioMérieux) at 37 °C in anaerobic atmosphere. Their identification was confirmed by the high scores (> 2) obtained using MALDI-TOF MS. In addition, 14 *S. intermedius* genome sequences were retrieved from GenBank. The 27 strains were divided into 8 groups according to their isolation source (Table 2). The genome sizes and gene numbers among *S. intermedius* strains were relatively similar, consisting for each strain in a single chromosome but no plasmid was identified in any strains and ranging in size from 1.85Mbp to 2.05Mbp (Table 2).

**Fig. 1:**
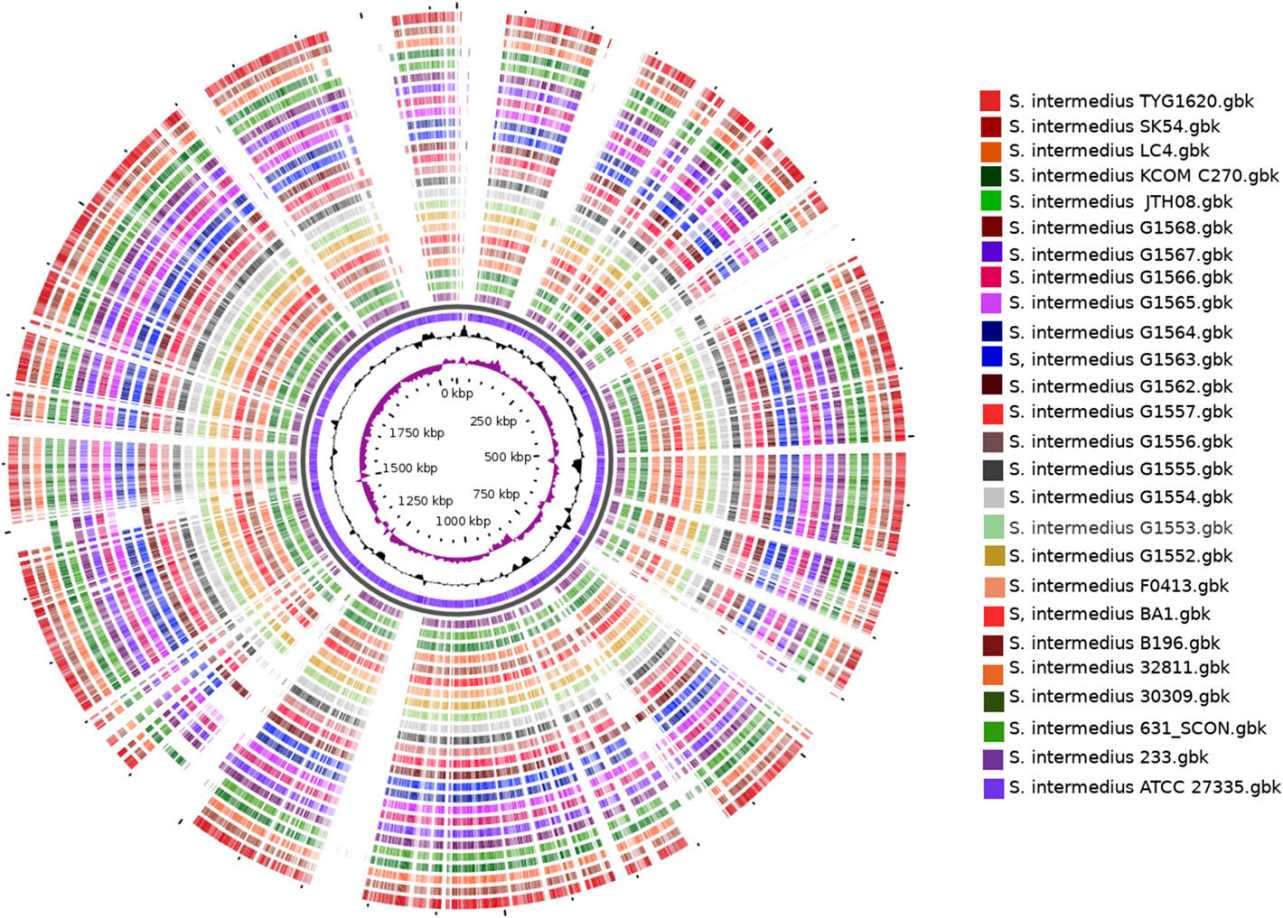
Circular representation of the 27 studied *S. intermedius* genomes. Genomic sequences were aligned using strain ATCC 27335 as reference. The alignment gaps tend to coincide with the regions of low G + C contents. The rings, from the inside out, display the size in kbp; GC skew; G + C content; followed by genomes as listed in the left legend

A schematic view of all 27 studied genomes is provided in (Fig. 1), showing an overall high degree of conservation. The general features of *S. intermedius* genomes are summarized in Table 2 The G + C content of *S. intermedius* ranged from 37.3 to 37.8 % (avg 37.641 %, *n* = 27). All 13 in-house sequenced *S. intermedius* contained at least 47 tRNA genes, and the number of rRNAs for all strains ranged from 3 to 6. *Streptococcus intermedius* exhibited an average 1870 CDs with a mean length of 907 bp, accounting for 87.3 % of the whole genome.

### Phylogenetic analysis

**Fig. 2:**
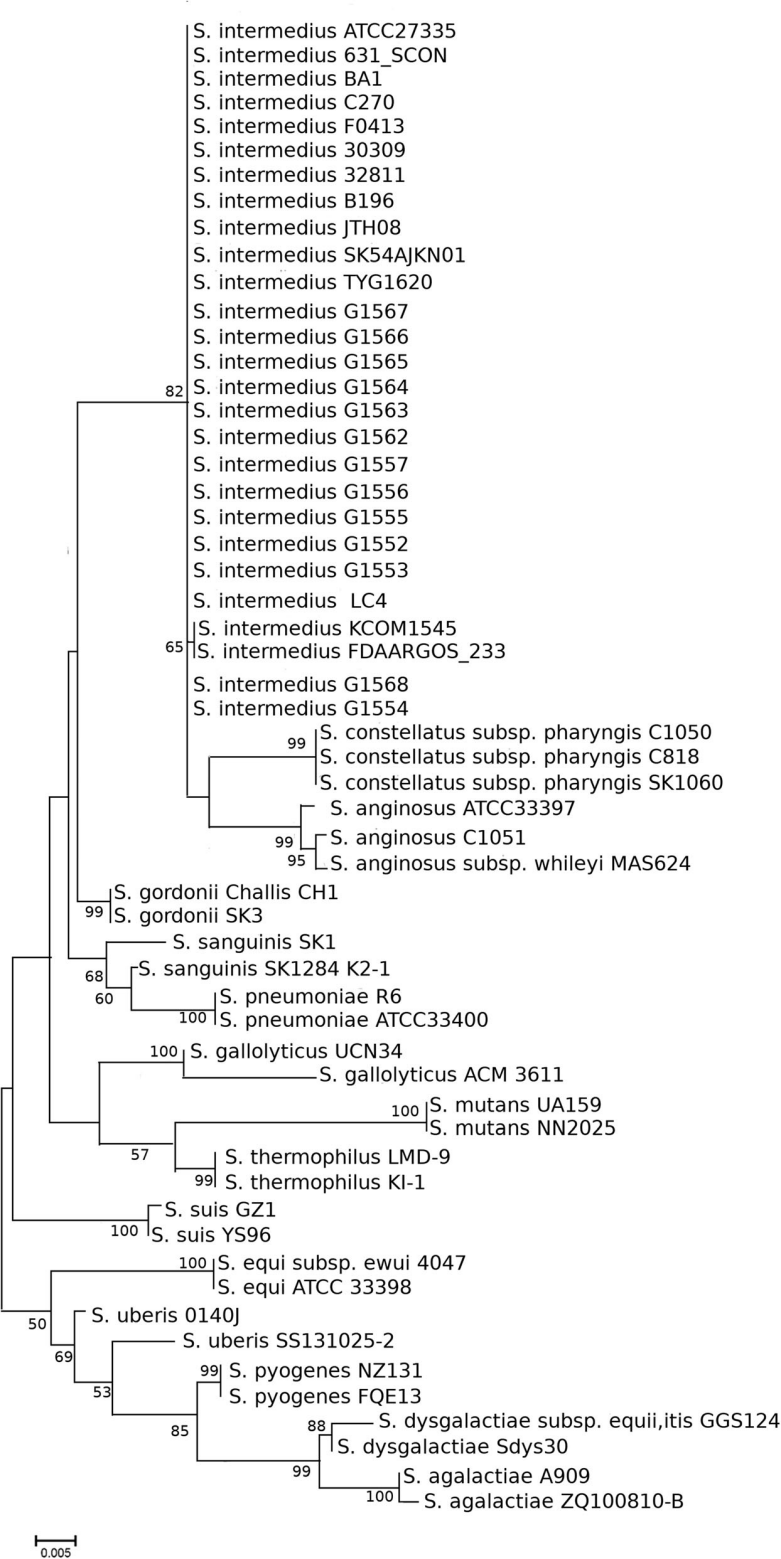
16S rRNA-based phylogenetic relationships of *S. intermedius* strains using the Maximum Likelihood method with Kimura 2-parameter. The scale bar indicates the evolutionary distance between the sequences determined by a 0.005 substitution per nucleotide position. Numbers at the nodes indicate bootstrap values obtained from 1,000 replicates

**Fig. 3:**
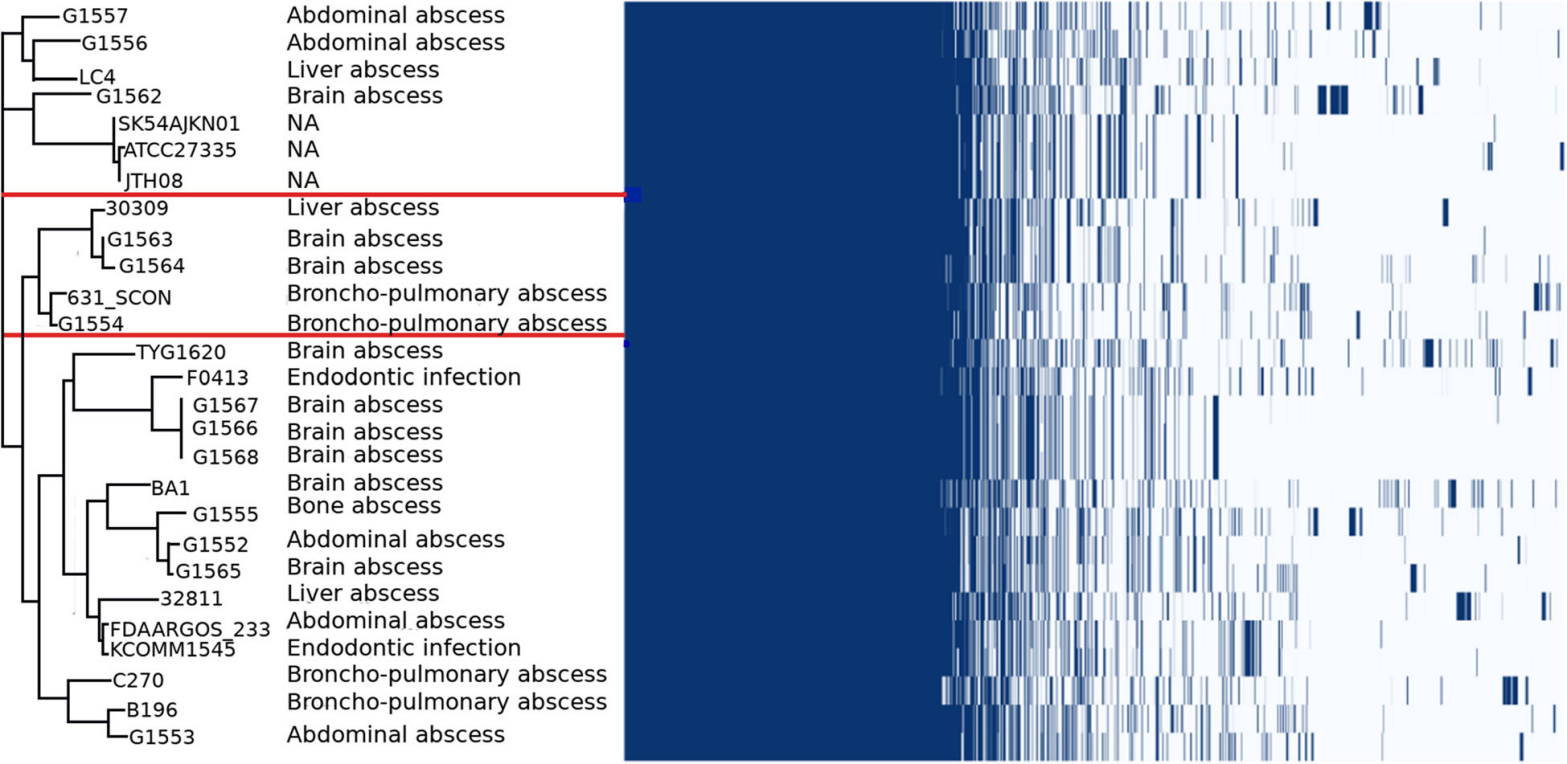
Clustered gene presence/ absence and accessory genome distribution calculated by pangenome analysis among the 27 studied *S. intermedius* strains. Left: core-genome phylogeny; the three clusters in the dendrogram are delineated by red lines; right: heatmap of core genes

The 16 S rRNA-based phylogenetic analysis (Fig. 2), widely used as a gene marker to differentiate *Streptococcus* species [34], demonstrated that all *S. intermedius* strains were grouped in a single cluster that was closely-related to *S. anginosus* and *S. constellatus* within the *S. anginosus* group [22] (Fig. 2). In the topology *S. intermedius*, *S. constellatus* and *S. anginosus* strains clustered together with their sub-species. However, the heatmap obtained using Roary [18], based on the core genome, was more discriminatory within the species than the 16 S rRNA-based analysis and identified 3 clusters that were independent from the strain source (Fig. 3).

The three clusters are as follows: strains G1557, G1556, LC4, G1562, SK54, AJKN01, ATCC27335 and JTH08 constituted the first group, strains 30,309, G1563, G1564, 631SC0N and G1554 clustered in the second group while the remaining strains clustered in a third group. There was neither evidence of correlation between strain clusters and their clinical forms, nor between genomic types and the geographical origin of isolates.

**Fig. 4:**
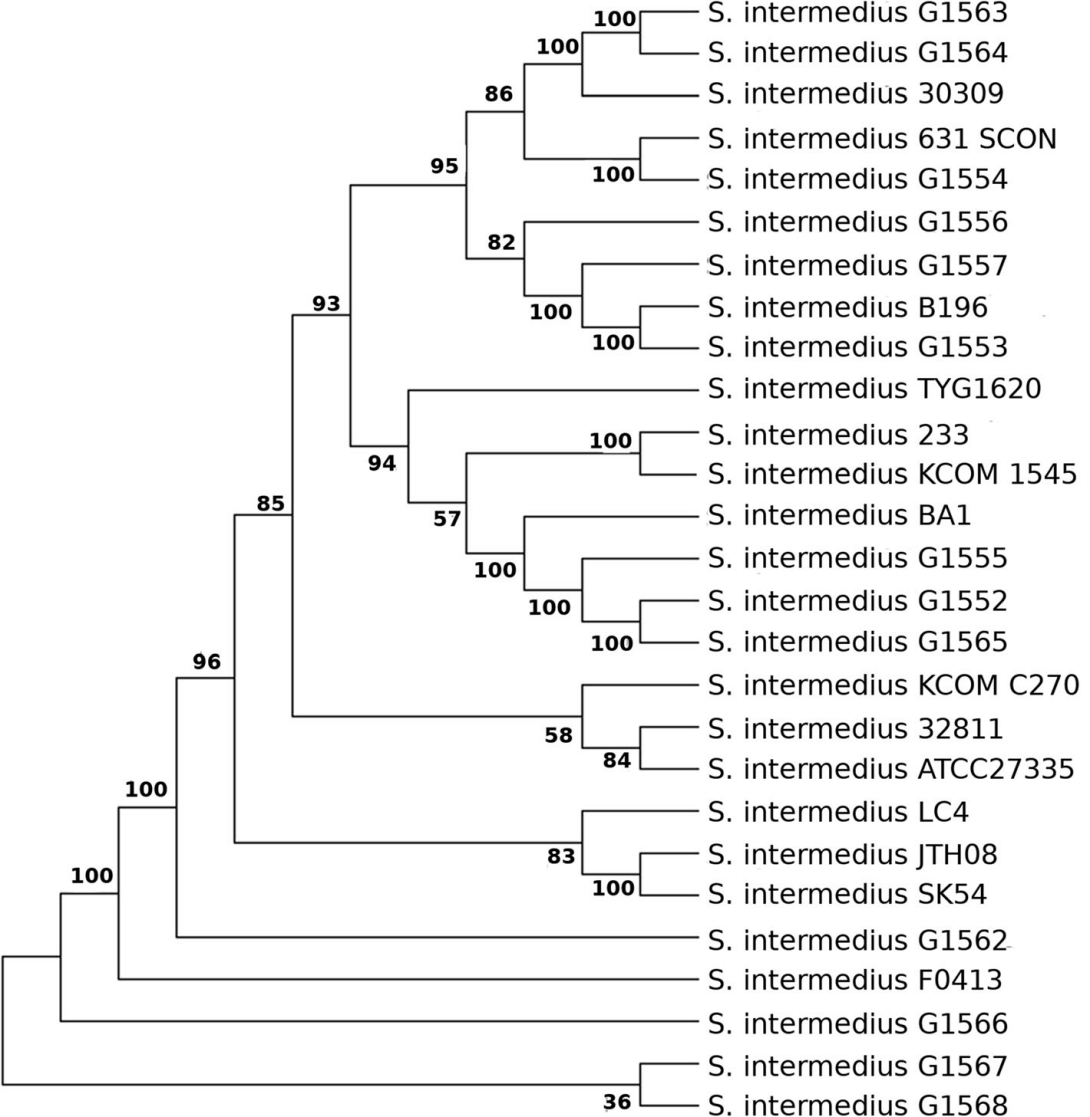
Phylogenetic tree of *S. intermedius* strains based upon SNPs extracted from the core genome. Sequences were aligned using ClustalW with default parameters and phylogenetic inferences obtained using the Maximum likelihood method within the MEGA, version 7, software. Nodes indicate bootstrap support from 1000 replicates.

To measure the divergence between all studied strains at a deeper level, we also analyzed their phylogenetic relationships on the basis of core genome SNPs, which demonstrated that strains G1562, G1566 and FO413 diverged from other strains and exhibited a higher tendency of recombination. However, again no disease-specific clustering was observed (Fig. 4).

### Genomic similarity

Digital DNA-DNA hybridization (dDDH) values ranged from 80.5 to 99.3 % between all 27 strains, thus confirming their classification within a single species. This was also cross-validated by the OrthoANI program, which produced pairwise values ranging from 97.78 to 100 % which is well above the consensus 95–96 % threshold for prokaryotic species demarcation [35]. This corresponded to 100 % 16 S rDNA sequence identity across all studied isolates. The above data correlate with a strong degree of genome conservation and synteny.

### Functional classification of orthologous cluster

**Fig. 5:**
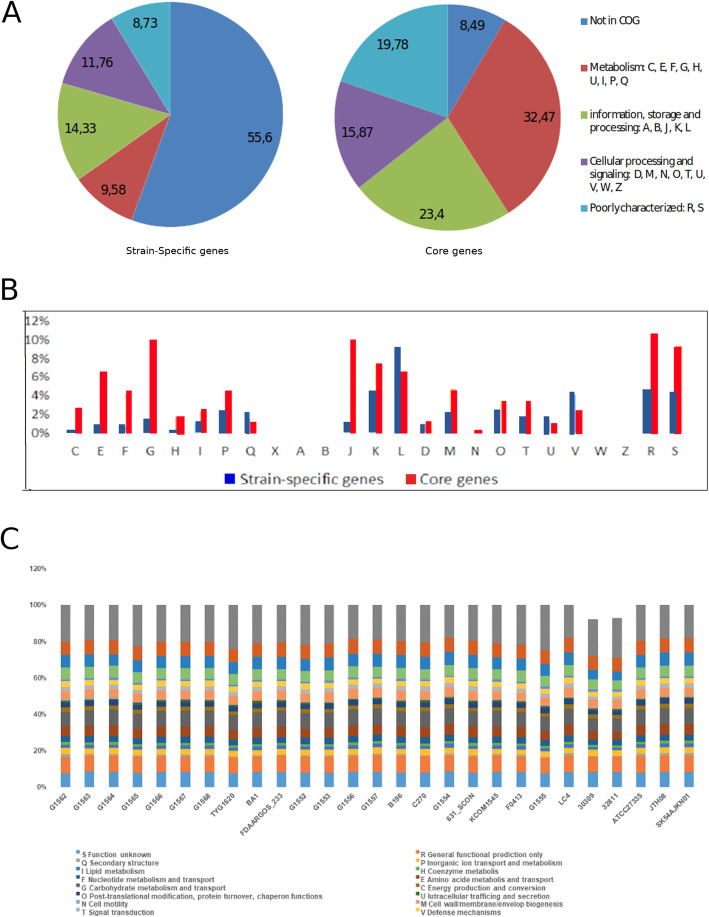
Differential distribution of COG functional categories in *S. intermedius*: **a** proportion of six classes of functional categories in strain-specific and core genes; **b** functional categories in strain-specific and core genes; **c** functional categories in the 27 *S. intermedius* strains. Category abbreviations are as follows: C, energy production and conversion; E, amino acid transport and metabolism; F, nucleotide transport and metabolism; G, carbohydrate transport and metabolism; H, coenzyme transport and metabolism; I, lipid transport and metabolism; P, inorganic ion transport and metabolism; Q, secondary metabolites biosynthesis, transport and catabolism; X, mobilome: prophages, transposons; A, RNA processing and modification; B, chromatin structure and dynamics; J, translation, ribosomal structure and biogenesis; K, transcription; L, replication, recombination and repair; D, cell cycle control, cell division, chromosomal partitioning; M, cell wall/membrane/envelope biogenesis; N, cell motility; O, posttranslational modification, protein turnover, chaperones; T, signal transduction mechanisms; U, intracellular trafficking, secretion, and vesicular transport; V, defense mechanisms; W, extracellular structures; Z, cytoskeleton; R, general function predicted only; S, function unknown

The overall distribution of *S. intermedius* proteins in COG categories was quite similar in all 27 studied strains (Fig. 5). Previous studies of other *Streptococcus* species also suggested that, within a given species, the majority of strains had a similar COG profile [36–38]. Approximately 79.72 % of all proteins predicted in all strains were identified in COG superfamilies. The proportion of each category fluctuated within a very small range, showing almost similar percentages of distribution in all strains. The most abundant sub-categories were related to carbohydrate transport and metabolism (G) and translation, ribosomal structure and biogenesis (J) like their distribution in core genes.

Less than half of strain-specific genes, but more than 90 % of core genes, had a match in the COGs database. The most abundant functions in core genes were associated with metabolism (Fig. 5a). The overall proportion of metabolic functions in core genes was 32.47 %, whereas that in strain-specific genes was 9.58 %. More specifically, energy production and conversion (C), amino acid transport and metabolism (E), nucleotide transport and metabolism (F), carbohydrate transport and metabolism (G) and coenzyme transport and metabolism (H) were noticeably more abundant in core genes (*p*-value < 0.01) (Fig. 5b). No mobilome-related functions were detected in *S. intermedius*. The functional category of information storage and processing showed highly different proportions in sub-categories (Fig. 5b). The functions of translation, ribosomal structure and biogenesis (J) were significantly enhanced (*p*-value < 0.0001) in core genes, whereas the functions of replication, recombination and repair (L) were significantly enhanced (*p*-value < 0.01) in strain-specific genes. This trend was also observed in other bacteria [35]. In the cellular processing and signaling category, the function of defense mechanisms (V) was more abundant in strain-specific (*p*-value < 0.05) than in core genes (Fig. 5c).

### Pan- and core-genome analyses

**Fig. 6:**
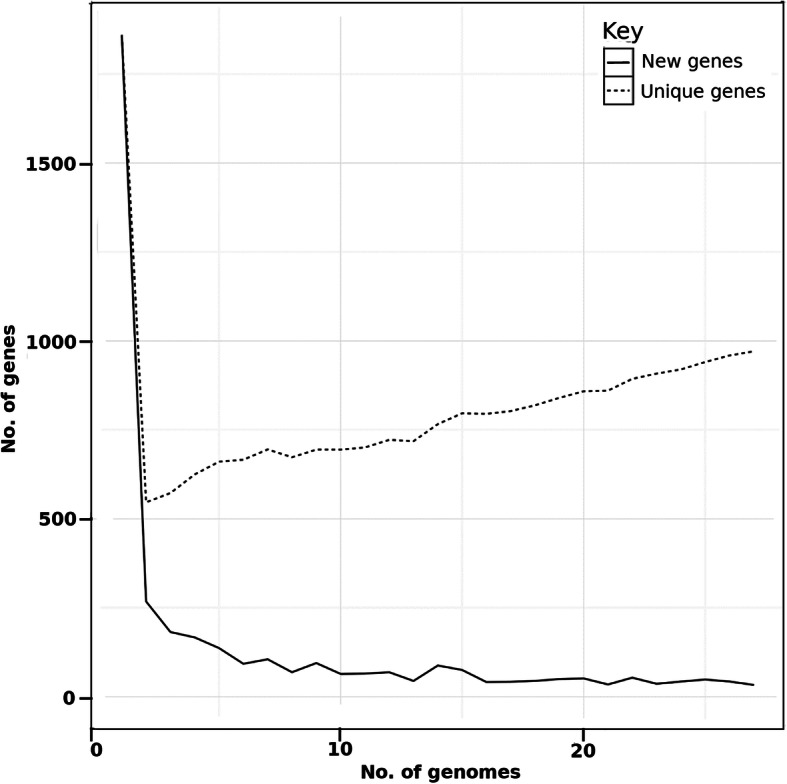
Plot representing the numbers of new and unique genes found as each isolate of *S. intermedius* is added

**Fig. 7:**
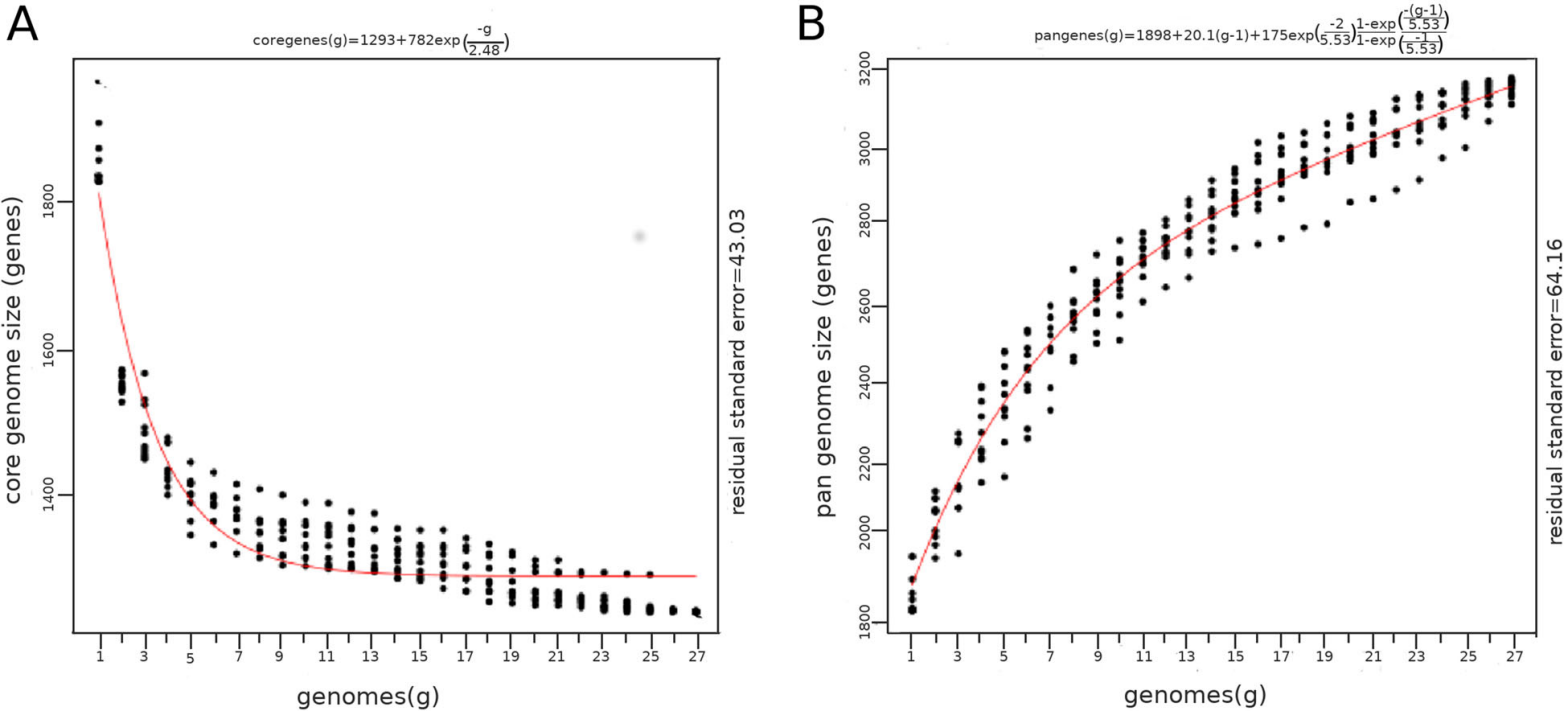
Fitted curves indicating the characteristics of core- and pan-genomes from 27 studied *S. intermedius* strains. **a** curve showing the relationship between the core genome and the number of genomes, **b** curve showing the relationship between the pan-genome and the number of genomes. As the number of genomes sequenced increased, the pan-genome size increased, whereas the core-genome size decreased, thus indicating an open pan-genome model. The gradual extension of the pangenome with addition of new genomes describes an open pan-genome model of *S. intermedius.* The number of genes that each strain contains is documented from comprehensive statistical analysis given earlier in Table 2

The average number of new genes added by a novel genome was 40 when the 27th genome was added (Fig. 6). The exponential decay model shown in Fig. 7a suggests that the number of conserved core genes approached an asymptote with the comparison of 27 genomes. A total of 1,355 core genes were identified in *S. intermedius*. The average proportion and sequence identity of core genes per strain were 72 and 97.79 %, respectively, indicating that core genes in *S. intermedius* are highly conserved and reflecting a low degree of intraspecies genomic variability too. Examination of the functional annotation of these core genes suggests, as expected, that they encode mostly core metabolic processes.

A total of 1,054 strain-specific genes were identified in *S. intermedius* and the average number of strain-specific genes was 39 (Fig. 7b). Among strain-specific genes, 148 genes were found in strain G1562, 107 in strain TYG1620, 105 in strain BA1, 96 in strain 32,811, 82 in strain G1557, 73 in strain C270, 69 in strain 631_SCON, 61 in strain G1555, 41 in strain G1554, 38 each in strains G1565 and F0413, 33 each in strains G1564 and LC4, 27 each in strains G1556 and ATCC27335, 20 in strain 30,309, 16 in strain G1553, 13 in strain G1552, 11 in strain B196, 6 in strain KCOM1545, 5 in strain FDAARGOS_233 and 1 in strains G1563, G1566, G1567, JTH08, SK54AJKN01, respectively. The size of the pangenome increased steadily without reaching any plateau. The pangenome trend depicted in (Fig. 7b) shows a gradual expansion by addition of new genomes and thus the pangenome of *S. intermedius* may be considered as open and indicates a homogenous pattern of genome evolution with similar rates of gene gain/ loss process across the whole population. In addition, a total of 1,611 accessory genes that were shared by two or more strains were identified. Overall, we identified a *S. intermedius* pangenome of 4,020 genes including 1,355 core genes, 1,054 strain-specific genes and 1,611 accessory genes.

### Virulence factors and principal component analysis

In the *S. intermedius* pangenome, 252 virulence factors were identified in total. Of these, 70 core virulence factors were shared by all strains and 78 unique virulence factors were present in one strain each (Table 3). Virulence-associated genes present in all studied genomes included homologous virulence genes that contribute to bacterial avoidance of the immune system, such as *ily* which encodes an intermedilysin, the *lmb, pspA, pav*B*/pfb*B, *fss*3 genes coding surface proteins, the genes coding the polysaccharide capsule (*cps*4A, *cps*4B, *cps*4C, *cps*4D, *cps*8D), the auto-inducer LuxS (*lux*S), the binding proteins (*pav*A, *hit*C, *fbp*C, *psa*A, *mnt*A, *clp*C, *fss*3), neuraminidase (*nan*A), hyaluronidase (*hys*A), and heat shock protein B (*htp*B), genes from the *sil* locus known to play a role in quorum-sensing and virulence in *S. pyogenes* (*sil*A, *sil*D, *sil*E, *sal*X), genes associated with secretion systems (*lem*11, *lem*15, *sde*C, *ceg*32, *esx*A, *ess*C, *lpg*2372, *lir*B), and genes associated with Mg2^+^ transport proteins (*mgt*B, *mgt*C); the response regulator *CsrR* beta-hemolysin gene *(cylG*), lamanin-binding surface protein like *Pac* and invasion protein *inlA* were also present in all strains.

**Table 3:** Unique Virulence-associated genes detected in the 27 studied *S. intermedius* genomes

Strain	Virulence factor	Amino acid identity%^a^
G1562	lpg2879	39.286
	*hlyD*	46.667
	*sdcA*	44.118
	*pvdD*	41.509
	*ybtE*	40.396
	*SalR*	37.879
	*legS2*	38.462
	*pieC/lirE*	36
	*pvcB*	38.776
	*inlK*	44.444
	*virB8*	38.235
	*sigA*	35.294
	*SalK*	36.585
	*pvdI*	37.092
G1563	-	-
G1564	*ctrD*	42.308
G1565	*aliA*	42.5
	*sspH2*	38.462
	*espN*	35.849
G1566	-	-
G1567	-	-
G1568	-	-
	*ipaJ*	38.298
	*FlgG*	40.909
	lpg1803	36.111
	*pilC*	39.286
	*brkA*	38
G1552	*pilT*	35.484
	*lbpA*	38.462
G1553	lpg0365	45.833
	*pvdJ*	36.667
	*ravI*	42.308
G1556	*vasL*	40
G1554	*flgK*	35.294
	*vopT*	52
	*iraB*	46.875
	*hopH*	41.935
G1557	*flgI*	39.286
	*toxB*	37.143
	*SalT*	35.294
	*lepA*	45.833
	*fcrA*	35.938
	MPN372	35.294
	*bepB*	36.364
	*wcbF*	35.714
G1555	*Iga*	35.556
	*lpsA*	36
	*flgJ*	36.957
	*stcE*	41.379
631_SCON	*Prt*	35
	*flgD*	37.143
C270	*eccE5*	36.111
	*lem7*	37.736
	EF0818	36.842
ATCC 27335	*vpdA*	38.462
JTH08	-	-
SK54AJKN01	-	-
32811	*Tox*	37.037
	*sipA/sspA*	38.235
	*fepA*	39.13
	*sdbC*	43.243
	lpg2525	50
30309	*flhA*	37.838
	*legC1*	38.235
F0413	*tlpB*	56.25
	*hopZ*	47.619
	*sidA*	42.424
	*hifB*	45.455
	*sipB/sspB*	35
LC4	*fliI*	36.364
KCOM 1545	-	-
B196	-	-
FDAARGOS_233	*srtD*	37.918
BA1	*zmpC*	54.839
	*recN*	42.105
	*bsc3*	47.826
	*vscN2*	36.364
	*fimC*	39.13
	*mavC*	35.593
	*fliF*	40
	*tcpB*	47.826
	*sfbI*	37.778
	*wcbN*	37.778
TYG1620	*pitB*	46.429
	*lem10*	57.143
	*lapB*	35.294
	*sopD2*	35.484

^a^Amino acid identity values were obtained by comparing each gene from each strain to the VFDB database [21]

Among these core virulence genes, the surface protein antigen I/II that was demonstrated to play a potential role in *S. intermedius* pathogenesis [39], and human fibronectin and laminin that are supposed to bind to this antigenic protein induce IL-18 release from monocytes [39]; genes from the streptococcal invasion locus (*sil*) are related to enhanced virulence in the SAG group and may contribute to the invasive behavior of *S. intermedius* strains; the internalin (*inlA*), likely acquired from *Listeria monocytogenes*, increases the virulence of *S. intermedius* by playing a key role in attachment to host cells [40]; the hyaluronidase (*hys*A) acts in the liquification of tissues and is also involved in biofilm formation, which protects bacteria from host defenses and antibiotics, and plays a role in infection [9]; the *ily*-coded intermedilysin can directly damage host tissues and immune defense cells, causing human cell death by membrane bleb formation [23]. It has been also reported that intermedilysin helps in invasion and adhesion of bacteria to human liver cells, and in cytotoxicity [41]; the *galE* gene codes galactose which plays a role in biofilm formation and its key residues are essential for epimerase activity [42]; the laminin-binding surface protein, homologous to that in *Streptococcus agalactiae* is coded by the *Pac* gene and is essential in binding and invasion of different host surfaces, and is present in almost all group B *Streptococcus* strains causing pneumonia, septicemia and meningitis [43, 44]; *psaA* codes a surface lipoprotein that plays a role in *Streptococcus pneumoniae* systemic infections by interacting with monocytes [45]; we also identified the heat shock protein-coding gene *htbB* that is known in *Legionella pneumophila* to act in adhesion to host fibronectin [46]; the *clpC* gene codes a heat shock protein involved in the invasion of hepatocytes in *Listeria monocytogenes* and has an ATPase activity [47]; ATPase proteins were shown to play a role in the survival and virulence in *Salmonella typhimurium* and *S. aureus *[48] ; *clpP* codes an ATP-dependent caseinolytic protease that was proven in *Streptococcus suis* to play a role in colonization and bacterial adaptation to various environmental stresses [49], *pavB* codes a fibronectin-binding protein that mediates bacterial attachment to human epithelial and endothelial cells and also promotes transfer of bacteria to the bloodstream [50, 51]; and *nanA* codes a highly conserved neuraminidase that also possesses a sialidase activity to catalyze the cleavage of terminal sialic acid residues from glycoconjugates. In *S. pneumoniae*, it promotes biofilm formation and contributes significantly to broncho-pulmonary colonization [52].

Although most of the strains exhibited one to eight unique virulence genes, strains G1562 and BA1 possessed 14 and 10 specific virulence genes, respectively. Eight strains (G1563, G1566, G1567, G1568, BA1, KCOM1545, JTH08, SK54AJKN01) had no strain-specific virulence factor (Table 3).

Among unique virulence genes, *sdcA, ybtE,lpbA, SalR,salK, VopT* are secretory system-associated genes that are involved in iron-mediated transport across cellular membranes. Some of these genes are linked with bacterial growth and act as important anti-inflammatory effectors [42, 53–58]. Among other unique genes, the *pilC* gene is suspected to be essential for secretion and assembly of transcription factor P, important in pilus formation [59] while *pilT* helps in polymerization and depolymerization of pilin [60]. The *brkA* gene inhibits bactericidal activity and protects the bacterium from complement activation products [61]. Other unique genes are linked with bacterial adherence and colonizationm such as *hopH, toxB, mpn 372* and *stcE* which contribute significantly to actin organization and bacterial attachment to human surfactant proteins [62–65]. The *iraAB* gene utilizes iron-loaded peptides that promote iron assimilation [66] while *lepA* plays a role in bacterial growth and induces inflammatory response. This gene also plays a key role in pathogenicity in *Psudomonas aeruginosa* [67]. The *fcrA* gene codes a protein containing receptor domains for immunoglobulins similar to those M-related proteins [68]. Another immunoglobulin-related gene, *aga*, plays a barrier function for mucosal antibodies by cleaving IgA1 [69]. *IpsA* controls transcriptional biogenesis of the cell wall in inositol-derived lipid formation in *Corynebacterium* and *Mycobacterium* species [70]. The *vasL* gene is considered to be component of *vas* genes, associated with the membrane type VI secretion system [71], and *ravL* is presumably activated at low oxygen level and regulates virulence gene expression via *clp* gene [72]. The *lpg0365* codes a lypophosphoglycan that together with other membrane polypeptides, is necessary for *Leishmania* pathogenesis [73]. The *pvdJ* gene is involved in the production of cyclodipeptides that may regulate the production of biofilm [74]. In addition, *pvdL* is associated to biosynthesis or uptake of the siderphores pyoverdine and pyochelin that act in the transport of heme and ferrous ions [75], while *pvdD* is involved in the biosynthesis of pyoverdine in *Pseudomonas aeruginosa* [76]. *IpaJ* codes a plasmid antigen involved in demyristoylation of proteins by inducing golgi fragmentation and inhibiting hormone trafficking [77]. *AliA* is associated with nasopharyngeal colonization in *Streptococcus pneumoniae* [78]. The *espN* gene is reported in *Mycobacterium tuberculosis* to play a role in adding an acetyl group to the N-terminus of the esaT-6 virulence factor [79]. Flagella-related unique genes found in different studied strains include *flgG, flgI, flgJ and flgk* which play a major role in virulence, adhesion and motility. They are mostly involved in flagellum formation and also act as interface with other flagellar proteins [80–83]. The *lnlK* gene was reported in *Listeria monocytogenes* to help avoid autophagy while *virB8* localizes to the inner membrane and is related to the export of alkaline phosphatase to the periplasm [84]. Finally, *sigA* codes a sigma factor linked with galactosidase activity [85].

**Fig. 8:**
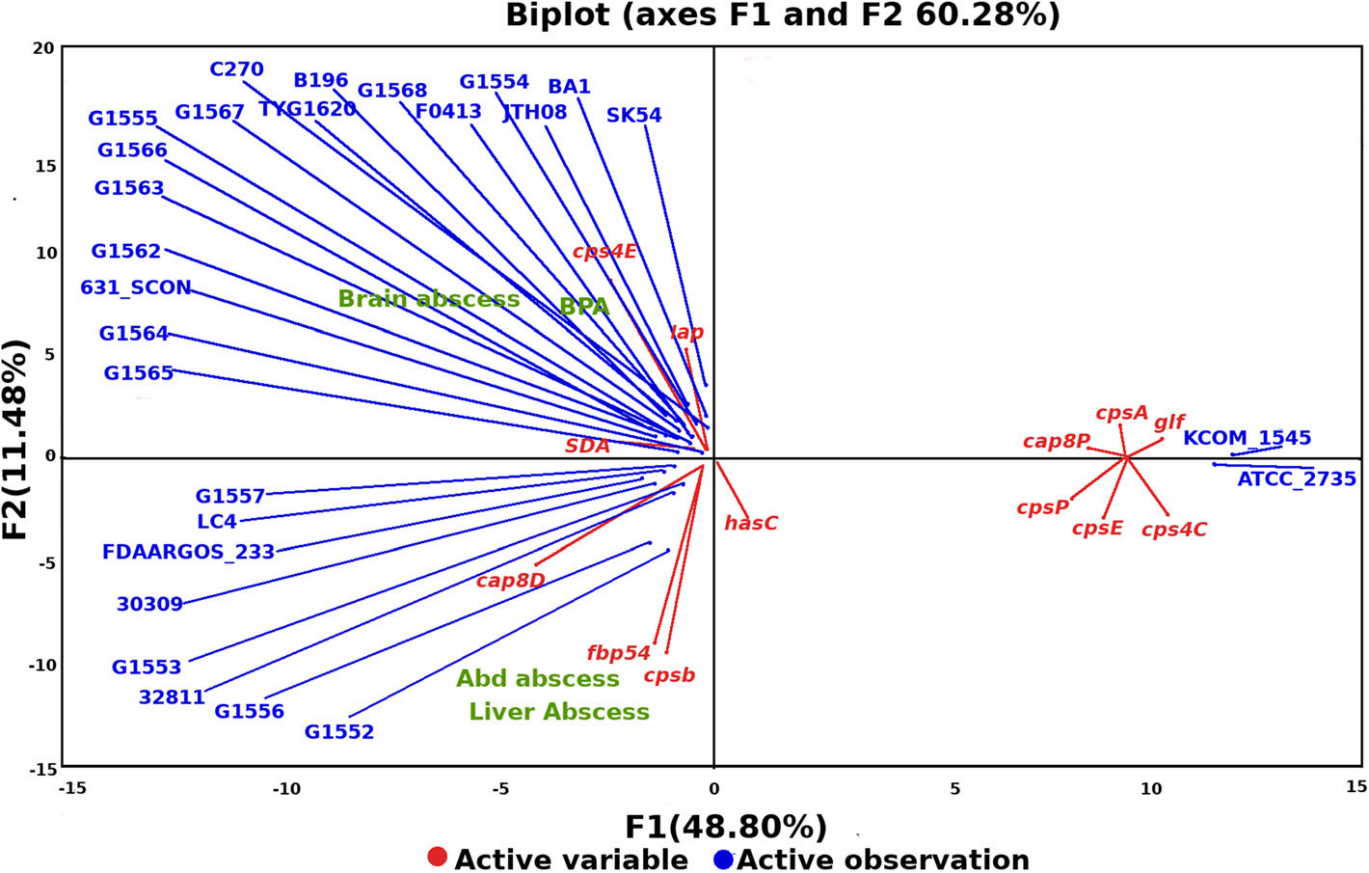
Principal component analysis based upon gene presence/absence showing the distribution of virulence genes which may contribute to the particular type of abscess. The green color represents the various clinical forms while virulence genes are represented in red and studied strains are in blue. BPA is Broncho Pulmonary abscess and Abd abscess denotes abdominal abscess

Using principal component analysis of differentially distributed virulence genes, three distinct clusters were visualized (Fig. 8). A clear separation of virulence genes associated with brain or broncho-pulmonary abscesses (*cps4E*, *sda* and *lap*) from those associated with liver or abdominal abscesses (*cpsB, fbp54* and *cap8D*) was observed. The first component which has maximum coverage and represents the largest variation showed that brain abscess-causing strains were associated with genes coding ATP-dependent proteolytic enzymes, which indicates their potential role in abscess formation. Other virulence genes clustered independently, excluding any association with the previous two disease categories. Among virulence genes associated to brain and broncho-pulmonary infections, *sda* codes an histidine kinase that regulates sporulation initiation in *Bacillus subtilis* and mediates the expression of virulence-associated factors [86]; *lap* codes the *Listeria* adhesion protein *(LAP)* that is a host stress response protein responsible for adhesion and promotion of translocation across monolayers [87]; and *cps4E* codes the capsular polysaccharide biosynthesis protein that was demonstrated in *S. pneumoniae* to prevent phagocytosis by forming an inert shield essential for encapsulation [88, 89].

In *S. pyogenes*, *fbp54* codes a fibronectin-binding protein that acts as an immunogen in humans. The amino acid sequence of *fbp54* in *S. intermedius* is similar to that of *S. pneumoniae. cap8D* codes a dehydratase that is essential for the synthesis of the capsule precursor involved in adhesion. It has also been targeted as component for vaccine development [85, 90]; *cpsB* code capsular polysaccharide biosynthesis proteins that are essential for encapsulation in *S. pneumoniae* and are involved in the interaction of bacteria with their environment, notably their host organism [91];

In contrast to the above-mentioned genes, some were not found to be disease-specific. These included *glf, cpsE, cpsI, cpsA, cps4C, cps8P* and *hasC*. The *glf* gene is involved in the biosynthesis of unusual monosaccharide galactofuranose [92]; *cpsE* codes a glycosyl transferase responsible for the addition of activated sugars to the lipid carriers in the bacterial membrane and are essential for encapsulation in *S. pneumoniae* [93]; *cpsI* is essential for the production of high molecular weight capsular polysaccharides [94]; *cpsA* and *cps8P* are necessary for normal cell wall integrity and composition [95]; *cps4C* codes a polysaccharide tyrosine kinase adaptor protein that plays a key role in the regulation of capsule biosynthesis [96]; finally, *hasC* is involved in biosynthesis of hyaluronic acid capsule biosynthesis encodes glucose-1-phosphate uridylyltransferase [97].

### Resistance-related genes and prophages

The tetracycline resistance gene *tet*M was identified in strains G1552, C270, KCOM1545, G1555, LC4, 30,309 and 32,811 whereas *tet*32 was identified in strain 631_SCON (Table 4). The macrolide resistance gene *ermB* was detected in strains G1552, C270, G1555 and 30,309. In other strains, no antibiotic resistance gene was identified.

**Table 4:** Antimicrobial resistance genes of studied *S. intermedius* strains

Strain name^a^	Resistance gene	Phenotype
G1552	*erm*(B)	Macrolide resistance
	*tet*(M)	Tetracycline resistance
C270	*erm*(B)	Macrolide resistance
	*tet*(M)	Tetracycline resistance
631_SCON	*tet*(32)	Tetracycline resistance
KCOM 1545	*tet*(M)	Tetracycline resistance
G1555	*erm*(B)	Macrolide resistance
	*tet*(M)	Tetracycline resistance
LC4	*tet*(M)	Tetracycline resistance
30309	*erm*(B)	Macrolide resistance
	*tet*(M)	Tetracycline resistance
32811	*tet*(M)	Tetracycline resistance

^a^Strains in which no resistance marker was detected were not included

A set of prophage elements was identified in all 27 strains (Table 1). In addition, four prophage-like elements were detected in strain BA1, three in strain TYG1620, and two in strains G1562, G1564, G1553, G1557, F0413 and G1555. The major difference in the genome size between all 27 studied strains of S. intermedius resided in the phage numbers and this presence of phages also denotes contribution of horizontal gene transfer in the emergence of this species [98].

### CRISPR identification analysis

The search for CRISPR elements showed that 14 of the 27 studied genomes contained CRISPRs. Three of these 14 strains (G1564, G1565, 631_SCON) had more than one CRISPR, for a total of 17 CRISPR modules identified in studied strains. The direct repeat (DR) length in identified CRISPRs ranged from 24 to 36 bp while there was variation in the number of spacers present within each CRISPR. CRISPRs also differed among strains but the DR regions were similar for a given CRISPR element subtype. Based on the type of cas proteins, the CRISPRs of strains G1562, G1563, G1564, G1556, G1554, 631_SCON, 30,309 were subtype I-C CRISPRs; those of strains FDAARGOS_233 and KCOM1545 were subtype II-A CRISPRs; finally, the CRISPRs of strains G1565, G1552, B196, G1555 and 32811were subtype II-C CRISPRs [93] (Table 5).

**Table 5:** CRISPR elements found in studied *S. intermedius* strains

Strain	DR length (nt)	Number of spacers	Spacer Length (nt)	CRISPR length (nt)	CRISPR type	DR consensus
G1562	32	23	33-37	1555	CAS-TypeIC	ATTTCAATCCACGCACCCGCGAAGGGTGCGAC
G1563	32	24	33-38	1632	CAS-TypeIC	ATTTCAATCCACGCACCCGCGAAGGGTGCGAC
G1564	32	17	33-38	1166	CAS-TypeIC	ATTTCAATCCACGCACCCGCGAAGGGTGCGAC
	32	6	33-35	429		ATTTCAATCCACGCACCCGCGAAGGGTGCGAC
G1565	36	33	29-31	2215	CAS-TypeIIC	GTTTTACAGTTACTTAAATCTTGAGAGTACAAAAAC
	36	9	20-30	628		GTTTTACAGTTACTTAAATCTTGAGAGTACAAAAAC
G1566	-					
G1567	-					
G1568	-					
TYG1620	-					
BA1	-					
FDAARGOS_233	36	17	29-30	1155	CAS-TypeIIA	GTTTTAGAGCTGTGCTGTTTCGAATGGTTCCAAAAC
G1552	36	23	29-30	1550	CAS-TypeIIC	GTTTTACAGTTACTTAAATCTTGAGAGTACAAAAAC
G1553	-					
G1556	32	6	33-35	427	CAS-TypeIC	GTCGCACCCTTCGCGGGTGCGTGGATTGAAAT
G1557	-					
B196	36	19	29-30	1288	CAS-TypeIIC	GTTTTTGTACTCTCAAGATTTAAGTAACTGCAAAAC
C270	-					
G1554	32	44	33-36	2950	CAS-TypeIC	ATTTCAATCCACGCACCCGCGAAGGGTGCGAC
631_SCON	32	1	33	96	CAS-TypeIC	GTCGCACCCTTCGCGGGTGCGTGGATTGAAAT
	24	1	32	79		ATGTACTTTATTTAAGTGAACACT
KCOM 1545	35	1	31	100	CAS-TypeIIA	GTTTTAGAGCTGTGCTGTTTCGAATGGTTCCAAAA
F0413	-					
G1555	36	38	29-31	2540	CAS-TypeIIC	GTTTTACAGTTACTTAAATCTTGAGAGTACAAAAAC
LC4	-					
30309	32	27	34-38	1830	CAS-TypeIC	ATTTCAATCCACGCACCCGCGAAGGGTGCGAC
32811	36	5	30	365	CAS-TypeIIC	GTTTTACAGTTACTTAAATCTTGAGAGTACAAAAAC
ATCC 27335	-					
JTH08	-					
SK54AJKN01	-					

## Conclusions

In the present study, we reported 13 new clinical isolates of *S. intermedius* and, based upon a combined approach of pangenomics, core-genomics and virulence profiling of 27 strains, attempted identification of disease-specific genetic profiles. The comprehensive analysis revealed a genomic variability across strains within the species, although synteny of the core genome was preserved. Our results highlight the importance of surface proteins like *pavB, pspA* and *cps4* (polysaccharide-coding proteins) and the binding proteins *psaA, pavA*, which are present in all studied strains, in pathogenesis. PCA results suggests two distinct categories of virulence genes, ATP dependent proteolytic virulence genes *cps4E, sda* and *lap* that are associated with brain and broncho pulmonary abscess while capsular polysaccharides protein coding genes *cpsB* and *cps8D* are linked with liver and abdominal abscess formation. The fibronectin binding protein coded by *fbp54* is also showing its connection with liver and abdominal abscess formation. A recent study also attempted to determine the pangenome of *S. intermedius*.[99] The SNP-based phylogenetic tree as well as core gene-based tree showed no clustering related to any disease entity in *S. intermedius* strains. The whole study provides a key genetic framework for assessing and understanding the molecular events contributing to *S. intermedius* pathogenesis. However, due to the limited number of studied strains, validation of the role of these virulence factors will require experimental confirmations.

## Data Availability

All Studied sequences are available in GenBank under accession numbers as follows: UENI00000000.1, UEND00000000.1, UENF00000000.1, UENG00000000.1, UICY00000000.1, UENA00000000.1, UENB00000000.1, AP014880.1, ANFT00000000.1, CP020433.2, UENJ00000000.1, NZ_UZBH00000000.1, UENK00000000.1, UENH00000000.1, CP003857.1, CP003858.1, UENC00000000.1, JUZI00000000.1, CP012718.1, AFXO00000000.1, UENE00000000.1, PNRP00000000.1, PNRI00000000.1, PNRH00000000.1, ATFK00000000.1, AP010969.1, AJKN00000000.1.
